# Reframing implementation science to address inequities in healthcare delivery

**DOI:** 10.1186/s12913-020-4975-3

**Published:** 2020-03-12

**Authors:** Ana A. Baumann, Leopoldo J. Cabassa

**Affiliations:** Washington University in St. Louis, Campus Box 1196, One Brookings Drive, St. Louis, MO 63130 USA

**Keywords:** Implementation science, Healthcare inequities, Adaptation, Equity

## Abstract

**Background:**

Research has generated valuable knowledge in identifying, understanding, and intervening to address inequities in the delivery of healthcare, yet these inequities persist. The best available interventions, programs and policies designed to address inequities in healthcare are not being adopted in routine practice settings. Implementation science can help address this gap by studying the factors, processes, and strategies at multiple levels of a system of care that influence the uptake, use, and the sustainability of these programs for vulnerable populations. We propose that an equity lens can help integrate the fields of implementation science and research that focuses on inequities in healthcare delivery.

**Main text:**

Using Proctor et al.’ (12) framework as a case study, we reframed five elements of implementation science to study inequities in healthcare. These elements include: 1) focus on reach from the very beginning; 2) design and select interventions for vulnerable populations and low-resource communities with implementation in mind; 3) implement what works and develop implementation strategies that can help reduce inequities in care; 4) develop the science of adaptations; and 5) use an equity lens for implementation outcomes.

**Conclusions:**

The goal of this paper is to continue the dialogue on how to critically infuse an equity approach in implementation studies to proactively address healthcare inequities in historically underserved populations. Our examples provide ways to operationalize how we can blend implementation science and healthcare inequities research.

## Background

Inequities in the delivery of healthcare are unjust differences between populations in the access, use, quality and outcomes of care [1]. Populations disproportionally impacted by these healthcare inequities include: racial/ethnic minorities, indigenous groups, socioeconomically disadvantage communities, and sexual and gender minorities, among others [2–8]. Inequities in the delivery of care are persistent, detrimental, and costly, and can no longer be ignored [1, 8].

The determinants of healthcare inequities are complex, multifactorial and involve the intersection of clients, family members, providers, healthcare organizations, and communities [6, 8, 9]. These inequities are perpetuated by the operations and ecology of the health care system, legal and regulatory climate, and discrimination and biases resulting in mistrust, stigma, lack of service affordability, and lack of cultural competence [1]. Inequities in healthcare are also rooted in the broader historic and contemporary social, economic, and political injustices that are present at all levels of care and service sectors throughout the globe.

The field of healthcare inequities research has generated valuable knowledge in identifying, understanding, and intervening to address inequities in care [2, 10]. Yet inequities in the delivery of healthcare persist. The field of implementation science can help address these inequities by studying the factors, processes, and strategies at multiple levels (e.g., clients, providers, organizations, communities) of a system of care that influence the uptake, use, and ultimately the sustainability of evidence-based interventions (EBIs) services, and policies in community settings [11–16]. The fields of healthcare inequities research and implementation science share common goals, such as striving to improve the quality and outcomes of services and making treatments and services generalizable to multiple communities and services sectors [13]. Both fields emphasize the importance of contextual factors in shaping these inequities and value multi-level approaches given the constellation of determinants linked to healthcare inequities.

Yet serious gaps in the integration of these fields remain. First, there continues to exist a persistent and stark underrepresentation of vulnerable populations in clinical trials informing the EBIs used in implementation studies [17]. The underrepresentation of vulnerable populations in clinical trials raises serious concerns about the external validity of these EBIs’, and points toward the need to reconfigure the process of intervention development for these historically underserved communities. Second, the broad implementation of EBIs often overlooks the importance of unique contextual factors that perpetuate inequities in healthcare. For instance, implementation efforts often ignore factors, such as economic, social, historical and political forces that shape the delivery of such interventions in low-resource settings and communities [8, 16, 18]. Third, the process of scaling up interventions developed in high-income countries to low-income countries often lacks the financial and human resources needed to implement these interventions [18, 19]. True partnerships with stakeholders from vulnerable contexts are not only ethical, but also enable the development of interventions and implementation strategies that are equitable for all [20].

In this paper, we propose that an equity lens can help integrate the fields of implementation science and healthcare inequities research. Equity focuses on providing what people, organizations or communities need to be successful. It requires that we move from treating everyone equally to proactively tailor the designs, methods, approaches, intervention, and implementation strategies to address the determinants of healthcare inequities. An equity lens forces us to break away from the “one size fit all” assumption and explicitly tackle the unique needs that vulnerable communities, settings and populations face in the receipt and delivery of healthcare [21].

Using elements of Proctor et al. [12] framework as a case study, we outline how implementation science can be reframed to study healthcare inequities. This framework differentiates between the intervention and their implementation strategies, and proposes three distinct but interrelated outcomes: implementation, service, and client outcomes [12]. We selected Proctor et al.’s framework because of its simplicity, its’ clear depiction of distinct and inter-related processes and outcomes, and because it is used to inform the design and evaluation of implementation studies [12]. We discuss five elements from the framework: 1) focus on reach from the very beginning; 2) design and select interventions for vulnerable populations and low-resource communities with implementation in mind; 3) implement what works and develop implementation strategies that can help reduce inequities in care; 4) develop the science of adaptations; and 5) use an equity lens for implementation outcomes. Table 1 outlines the summary of these elements and our recommendations for research. Our intent is not to develop a new framework. A plethora of implementation science frameworks already exist [22]; what is needed are more examples on how to apply existing frameworks to address public health issues, such as inequities in healthcare.

**Table 1:** Reframing elements of implementation science to address inequities in healthcare delivery

	Key points	Recommendations
Focus on reach from the very beginning	• The underrepresentation of vulnerable populations and communities persist in clinical and implementation trials. • Equity requires attention to reach and representation in clinical trials and implementation studies • Studies need to mirror the context where vulnerable populations are served and live.	• Increase enrollment and engagement of vulnerable populations in clinical and implementation trials • Broaden the settings and communities where implementation studies are conducted
Design and select intervention for vulnerable populations with implementation in mind	• The linear process of intervention development contributes to implementation gaps. • An implementation perspective to intervention development forces developers to consider the fit between the intervention and the implementation context.	• Place implementation outcomes at the forefront of the intervention development process • Incorporate user-centered designs and participatory approaches to develop interventions *with, for* and *in* the community.
Implement what works and develop implementation strategies that can help reduce inequities in care	• Evidence based interventions (EBIs) known to reduce inequities in care are not routinely used in real-world settings. • Implementation strategies can support the adoption of EBI in vulnerable communities.	• Invest in the identification, development, and testing of implementation strategies for EBIs that can reduce healthcare inequities. • Implementation strategies in vulnerable communities may need to include additional components (e.g., cultural competence, advocacy)
Develop the science of adaptation	• Attention to the unique contextual factors that influence healthcare inequities is critical to implement EBIs in vulnerable populations. • A broader conceptualization of adaptation is critical for addressing healthcare inequities as it expands the purview of adaptations to consider the EBI, implementation strategies, and the context of practice.	• Adaptations need to be done systematically and be guided by frameworks. • A common data platform can be used to track and help identify optimal adaptation across different contexts and populations. • Adaptations can be used as an implementation strategy.
Use an equity lens for implementation outcomes	• Implementation outcomes are important because they are interrelated with services and client outcomes. • Limited attention has been given to examining issues of equity in implementation outcomes.	• Descriptive and explanatory studies are needed to identify the factors and mechanisms that contribute to inequities in implementation outcomes • Conduct studies to develop, test, and refine implementation strategies to achieve equity in implementation outcomes.

### Focus on reach from the very beginning

Equity requires thoughtful attention to reach and representation; who is invited and included, who is participating and engaged, and who is missing from our implementation studies [23, 24]. Reach is a long-standing problem when it comes to the inclusion of vulnerable populations in the clinical trials that inform the development of EBIs used in implementation science. For example, a review of randomized clinical trials conducted in the U.S. between 2001 to 2010 for common mental disorders showed that racial and ethnic minorities were seriously underrepresented, accounting for 19% of the total sample in these trials; with some populations being represented less than 1% (e.g., American Indians, Alaska Natives) [17]. Despite major efforts to address this long-standing problem, the underrepresentation of vulnerable population persists in the U.S. [17, 25] and globally [26]. This same problem is present for other health conditions (e.g., prostate cancer) [27–29].

The exclusion of historically underserved communities in clinical trials creates serious blind spots not only in treatment science but also in moving this science into practice. The evidence from efficacy and effectiveness trials is used to develop treatment guidelines and standards of care. Given the underrepresentation of vulnerable populations in these trials, the generalizability of guidelines and standards of care is questionable, particularly when the EBIs are implemented in routine practice settings. The absence of these populations in the generation of evidence of intervention effectiveness is a common limitation in implementation studies as it results in the adoption of interventions that often neglect the unique needs and context of these communities [18, 25].

There are several solutions to address the problem of reach from an equity perspective. Central to an equity lens is to increase representation in the enrollment and engagement of vulnerable populations participating in clinical trials. A growing literature provides strategies for recruiting diverse populations into clinical trials. For example, studies have shown the importance of building relationships and trust [30], including family referrals and community stakeholders [31], person-to-person outreach, presentations in the community, and face-to-face meetings [32]. More recently, studies have evaluated the use of popular social media platforms (e.g., Twitter, Facebook [18, 33]) in recruiting vulnerable populations.

Moving beyond clinical trials, reach in implementation studies is critical. Given the reality that vulnerable populations tend to be absent from clinical trials, implementation scientists need to examine where the populations from our implementation trials are, where they are served, and who is providing services to them. For example, conducting studies in non-traditional settings, such as faith communities, family resource agencies, barbershops, and community centers, can increase reach because vulnerable populations may not go to traditional healthcare settings due to stigma, mistrust and discrimination [30, 34–37]. Implementation and health inequity researchers should aspire to conduct studies that mirror the context of vulnerable populations.

### Design and select interventions for vulnerable populations with implementation in mind

The traditional linear process of intervention development, from proof of concept and efficacy, to effectiveness and implementation trials contributes to the implementation gap [10, 38]. This process faces an inherent “disconnect between the design context and the implementation context” which leads to treatments that are often not feasible, acceptable, and useful in communities that are under resourced, and do not fit with the realities of routine practice [39]. The disconnect between the intervention and context may be more pronounced in historically underserved communities, thus requiring an additional step to adapt EBIs to vulnerable populations. This extra step in the linear process of treatment development is necessary and important, but it can create further delays in translating research into practice [10].

Reconfiguring the treatment development process for vulnerable populations could benefit from designing and selecting interventions with the end in mind. This perspective forces the intervention developer to consider the fit between the intervention and implementation context from the very beginning. Designing with implementation in mind requires placing implementation outcomes, such as acceptability, feasibility, appropriateness, and cost, at the forefront of the intervention development process [12]. These outcomes need to be examined and triangulated from multiple stakeholder perspectives: clients, family members, providers, administrators, community leaders and policy makers. One methodology that applies this perspective is user-centered designs, commonly used in the development of consumer products [39–43].

Another element of developing interventions with implementation in mind, especially for vulnerable populations, is to conduct the intervention development process *with*, *for,* and *in* the community, bringing science and practice closer together. Bridging science and practice requires a collaborative lens from the very beginning. Community-based participatory research (CBPR) is one approach that focuses on fostering partnerships between stakeholders by capitalizing on their shared and local knowledge, wisdom, and expertise [44]. CBPR helps to: (a) contextualize interventions to the realities and conditions of specific communities and settings; (b) integrate social and cultural values, perspectives, and norms into the development and implementation of interventions to enhance their relevance, acceptability, and effectiveness; and (c) build capacities of stakeholders to produce community-engaged research and practices critical for reducing inequities in healthcare [37, 45, 46]. Hopefully these methodologies will help develop interventions that are meaningful and more effective for vulnerable populations.

### Implement what works and develop implementation strategies that can help reduce inequities in the delivery of healthcare

A growing literature supports the effectiveness of some EBIs for reducing inequities in healthcare delivery [47–50]. Unfortunately, the state of health inequities research is to continue testing the effectiveness of EBIs for different populations and contexts; getting stuck in a loop between efficacy and effectiveness trials [10]. The result of this loop is that these EBIs are rarely implemented in community settings serving vulnerable populations [9, 10, 13, 14, 17, 18].

To break the efficacy/effectiveness loop for EBIs that have mature evidence for addressing healthcare inequities, researchers need to focus on their implementation in the community using implementation strategies. These are systematic and planned processes and actions that are designed to help integrate EBIs’ into practice settings [51]. Implementation strategies come in many forms, such as discrete single actions (e.g., training workshops), multifaceted approaches (e.g., training workshops with supervision and fidelity feedback), or blended methods (e.g., learning collaboratives) [51, 52]. Implementation strategies are used “to plan, educate, finance, restructure, manage quality, and attend to the policy context to facilitate implementation” [53].

There are two areas of research for implementation strategies of EBIs that can address healthcare inequities. First, we need to identify and develop the implementation strategies that can accompany these EBIs. An important source of information for the development of implementation strategies can come from what is already being done in existing effectiveness trials such as how we train, supervise, and track intervention fidelity. We can also draw from existing compilations of implementation strategies [52, 53]. Similar to the formative research done to develop and adapt EBIs, we can do formative evaluations to understand the factors and processes that shape how to get these EBIs into practice [54]. This process needs to be done systematically, theoretically-informed and grounded in addressing the unique contextual determinants of healthcare inequities. The ideal result recommended by implementation scientists are the creation of implementation strategies that specify the actors involved, the steps, frequency, and actions that need to be enacted, what needs to be changed, and what are the expected outcomes [55].

Second, we need to test the effectiveness of these implementation strategies. For example, conducting a randomized controlled trial testing two modes of implementation of the same EBI (e.g., online vs. in-person). Considering that vulnerable populations often receive services in low-resource settings, additional components and processes may be needed to support the implementation of EBIs in these communities such as, attention to financial resources, definition of roles and responsibilities, building and sustaining local capacity, restructuring the delivery of care, having leadership support and staff buy-in, among others [56, 57]. Additionally, because of the multiple economic, political, and historical forces that shape the delivery of EBIs in low-resource communities, implementation strategies for EBIs that focus on vulnerable populations need to consider the multifactorial determinants of healthcare inequities. Researchers may need to include components that engender trust in the community, enhance cultural competence, raise critical consciousness, support advocacy, and reduce language barriers, [45, 57–59].

### Develop the science of adaptations

Implementation is about the fit between the EBI and the context of practice. Based on the diffusion of innovation theory, an “[EBI] almost never fits perfectly in the organization in which it is being embedded”, suggesting that adaptations to the intervention and/or the context of practice are critical to the implementation process [60]. Adaptations are thoughtful modifications made to the intervention with the goal of improving their fit with a given context [61, 62]. Multiple models and methods have been developed and used to inform the adaptations of EBIs [63–65]. Systematic literature reviews and meta-analyses support the importance of adapting EBIs to improve outcomes, particularly in vulnerable populations [66–69].

Most adaptation models and efforts focus exclusively on modifying the intervention [21]. However, the implementation strategies and the context in which the EBI is being implemented may need to be modified. Attention to the unique contextual factors that influence healthcare inequities is critical to enhance the uptake and the ultimate impact of EBIs in vulnerable populations [70–72]. Implementation requires not only changes to the EBI but also to the context of practice, such as thoughtful modifications to provider, organization, and community factors [18, 44]. This broader conceptualization is critical for addressing healthcare inequities as it expands the purview of adaptations to consider the EBI, the implementation strategies, and the context in which the implementation process is taking place, thus enhancing the chances that the EBI will be integrated into community settings.

Several steps are needed to develop the science of adaptations supporting this broader conceptualization. First, adaptations need to be done systematically and follow established practices to avoid intervention drift [73]. Common practices in adaptation frameworks include: (a) involvement of stakeholders (e.g., researchers, clients, providers, administrators), including collaborations between treatment developers, treatment users, and community members; (b) use of formative and participatory research methods to understand population needs, risk and resilience factors, and the context of practice; (c) systematically document the process of engagement and retention of participants, providers and organization; and (d) follow a step-wise process moving from formative to evaluation research addressing the fidelity-adaptation tension via iterative pilot testing to refine the intervention and its implementation strategies [44].

Second, a common data platform should be used to systematically track adaptations [74–76]. Standardizing the tracking process of modifications done to the intervention, implementation strategies and to the context will help us build the knowledge base of what is being adapted, who is adapting, how, when, and why adaptations are being made [76]. Documenting adaptations can be done through a variety of methods, such as direct observations, checklists, key informant self-reports, semi-structure interviews, focus groups, review of charts, intervention manuals, project documents, and/or meeting minutes [62, 74]. Tracking can occur as adaptations are being planned, in-real time, and/or retrospectively [76]. As with any emerging science, further work is needed to examine the reliability, validity, burden, and cost of these tracking methods and how to make them more pragmatic, particularly for low-resource settings [77].

The accumulation of this evidence will help develop what Chambers and Norton [73] have called the ADAPTOME: “a systematic and robust body of knowledge that chronicles the many types of adaptations to [EBIs] and their impact on implementation, service and health outcomes.” [73] The creation of the ADAPTOME will help facilitate the understanding of associations between the processes, types, and reasons that interventions and implementation strategies are being adapted and the impact that adaptations have on implementation, services and client outcomes. This data platform is critical for addressing healthcare inequities because it will allow us to empirically comprehend the optimal adaptations that are needed across different contexts and populations.

Third, while much has been written about adaptations to interventions and its promise to decrease healthcare inequities [78–80], less is known about how adaptations can facilitate the implementation of EBIs in historically underserved populations. In our broader conceptualization, adaptation can be considered an implementation strategy because it addresses the fundamental interplay between the EBI (the *what*), the process of implementation (the *how)*, and the context of practice (the *where*). The Dynamic Adaptation Process (DAP) developed by Aarons and colleagues [81] is an example of adaptation as an implementation strategy. The DAP is a planned and systematic approach that uses an implementation resource team composed of multiple stakeholders (e.g., researchers, intervention developers, administrators, clinicians, clients) working together to identify core and adaptable elements of the EBI being implemented, and assessing system-, organization-, provider- and client- levels characteristics that need to be considered for effective implementation. This group also supports the implementation process via ongoing training and coaching, fidelity monitoring and real-time problem solving and adaptation to the context of practice.

In all, implementation is all about fit. Nothing can be implemented without some level of mutual adaptation to either the EBI and/or the context of practice. Further understanding of adaptation as an implementation strategy is needed in the field to be able to replicate and test its efficacy and determine its generalizability in different contexts and populations.

### Use an equity lens for implementation outcomes

The aim of equity in healthcare is that all people are treated fairly by the systems of care based on their needs, not on their personal backgrounds [1]. It requires fair access to high quality healthcare to achieve optimal health, functioning, and well-being [14]. An equity lens focuses on providing what people, organizations or communities need to be successful by tailoring the designs, methods, approaches, intervention, and implementation strategies to address the determinants of healthcare inequities. This approach forces us to examine the social, political, and environmental context of the intervention and implementation strategies that are being delivered in implementation studies to evaluate and address inequities in healthcare delivery. Traditionally, efforts to address inequities in healthcare have focused on Proctor’s et al. [12] services- (e.g., efficiency, effectiveness, timeliness) and client- (e.g., satisfaction, functioning, health status) level outcomes among specific historically underserved populations. More investments in research are needed to address inequities with an emphasis on eliminating gaps in these outcomes between vulnerable groups and their more advantaged counterparts [2, 10].

In addition to service and client outcomes, Proctor et al. [12] identified implementation outcomes, defined as “the effects of deliberate and purposive actions to implement new treatments, practices, and services” [12]. Implementation outcomes are essential for evaluating the success of implementation efforts because if an EBI is not implemented correctly, it will not attain its desired goals [12]. Less attention has been given to examining issues of equity in implementation outcomes. We know little about potential inequities in outcomes, such as feasibility, fidelity, penetration, acceptability, sustainability, uptake, and cost of EBIs when implementing them in routine practice settings serving vulnerable populations.

There are two possible interrelated lines of research to understand and address the intersection of implementation outcomes and equity. First, the field of implementation science needs descriptive and explanatory studies to identify the variety of factors and mechanisms that may contribute to inequities in implementation outcomes. Potential questions in this area could be: what community-, organization-, provider- and client-level factors contribute to inequities in implementation outcomes between organizations delivering the same EBI to different populations? Do organizations that serve large populations of racial/ethnic minorities achieve the same implementation outcomes (e.g., fidelity, cost, sustainability) as those that serve predominantly non-Hispanic Whites?

A second line of research moves from description to action applying the knowledge generated from the studies described above to develop, test, and refine implementation strategies to achieve equity in implementation outcomes. Potential questions in this area could be: Which implementation strategies produce equitable implementation outcomes between organizations delivering the same EBI to different populations? How to adapt implementation strategies for organizations serving vulnerable populations to achieve equity in implementation outcomes? Because implementation outcomes are interrelated with service and patient outcomes, it is our hope that using an equity lens when examining implementation outcomes will contribute to the reduction of inequities in the delivery of healthcare.

##  Discussion

Inequities in healthcare continue to persist despite important advances and investments in research, policy reforms and system redesign. In this paper, we discussed how an equity lens can be applied to implementation science to help address inequities in healthcare. We illustrated how to reframe five elements of an existing framework, Proctor et al. [12], to position equity at the forefront of implementation studies.

First, we discussed the importance of focusing on reach from the very beginning as we conceptualize and initiate effectiveness and implementation studies. Equity demands that our studies include the communities that are most at need for high quality healthcare. We can achieve a larger reach of participants by broadening where we conduct our studies, who we invite and enroll from our communities. This requires federal agencies, state and local governments, and private foundations to invest in ending the historical underrepresentation of vulnerable populations in intervention and implementation studies. In addition to funding opportunities, grants and contracts need to be restructured to build time, resources, and infrastructure in the initial stages of a project to develop true partnerships between researchers and vulnerable communities. Concerted reforms are also needed to value and include community-engaged research in promotion decisions in academic and research institutions.

Moving beyond reach, we discussed the importance of designing and selecting intervention for vulnerable populations with implementation in mind. The goal of this approach is to increase the fit between the intervention and the context in which it is to be delivered, thus bringing research and practice closer together. This requires a break from the traditional linear approach of intervention development. It embraces a reconfiguration of this process in which interventions are developed, tested, refined, and implemented from the very beginning with the community using collaborative and participatory processes. For this reconfiguration to be successful, collaborations between all the stakeholders involved in an intervention, from the developers to the clients, including implementation scientists, is key.

For those interventions known to reduce healthcare inequities, we need to rigorously identify, develop, and test implementation strategies that can support their use in the community. More investments in implementation studies are needed to help break the efficacy/effectiveness loop inherent in healthcare inequities research [10]. An important area of investigation is to identify which existing implementation strategies facilitate the implementation of EBIs to reduce healthcare inequities. Given the unique contexts that perpetuate inequities in care, implementation strategies may need to include components such as building community trust, enhancing cultural competence, raising critical consciousness, supporting advocacy, and reducing language barriers [34, 36, 45, 82].

Furthermore, we proposed the development of a science of adaptation to examine the modifications not only to the intervention, but also to implementation strategies and the context where the implementation process is taken place. As such, context is brought at the forefront and embedded as part of the process instead of being a “nuisance” in our studies [83]. To be able to foster the science of adaptation, we propose standardization, planning and careful documentation of the modifications done as we move along the implementation process. Our team and others have proposed methods and frameworks to track adaptations [74]. The accumulation of this information will allow for the creation of a database and further understanding of the associations between the processes, types, and reasons for adaptations and the impact that these modifications have on implementation, services, and client outcomes [73].

Our final point focuses on implementation outcomes which are interrelated with service and patient outcomes. Little is known about inequities in implementation outcomes. To address this gap, we recommend using an equity lens to help identify and understand potential inequities in implementation outcomes. By delineating inequities in implementation outcomes, the implementation science field will be in a better position to develop and test implementation strategies that will address healthcare inequities.

### Considerations for global inequities in healthcare delivery

The ideas outlined in this paper may have greater applicability to the U.S. and may not directly fit to other countries. However, inequities in the delivery of healthcare are a global issue. Ongoing efforts are documenting the magnitude of these healthcare inequities, identifying interventions to improve healthcare delivery, and improving cross-country communications for better implementation of these interventions for communities in need [84]; e.g., [85, 86]. A common challenge in these efforts is finding the balance between a global response to address inequities in healthcare delivery while respecting and understanding the unique systems of care across countries. First, healthcare systems and the inequities in the delivery of care that exist within these systems vary considerably across the globe. Second, clinical trials in low- and middle- income countries are often carried out with different controls required in the global North. Often, the research guidance and oversight for clinical trials vary across countries, posing a conundrum in terms of ethics and equity in clinical research [87]. Thus, the findings of these trials should be taken cautiously when implementing across countries. Third, clinical trials tend to be highly concentrated in high-income countries compared to low-and-middle income countries [88]. Therefore, the arguments outlined in this paper should be taken with caution when considering the global context.

## Conclusions

In conclusion, the goal of this paper is to continue and deepen a much-needed dialogue on how to critically infuse an equity approach in implementation studies to proactively address healthcare inequities in historically underserved populations. The fields of implementation science and healthcare inequities could benefit from uniting their perspectives.

## Data Availability

Not applicable.

## Abbreviations

CBPR: Community-based participatory research (CBPR)
DAP: Dynamic Adaptation Process
EBI: Evidence Based Intervention
U.S.: United States
